# Medical education during pandemics: a UK perspective

**DOI:** 10.1186/s12916-020-01577-y

**Published:** 2020-04-09

**Authors:** Areeb Mian, Shujhat Khan

**Affiliations:** Department of Medicine, Imperial College London, London, SW7 2AZ United Kingdom

**Keywords:** COVID-19, Medical education, Pandemics, Teleteaching, Telemedicine

## Introduction

As the coronavirus (COVID-19) pandemic becomes widespread, its impact on society is becoming more pervasive and is now threatening medical education. Numerous medical schools have suspended all clinical placements and classes with the hopes of mitigating viral transmission. The timing of this disruption is set to have profound consequences as universities, particularly in the UK, are now conducting assessments remotely, and some are considering deferring students due to the inability to carry out teaching and clinical placements. Here, we discuss the different modes of teaching that may be offered during this time.

Over the last several years, some medical schools have shifted from traditional forms of ‘in-person’ lecture-based teaching to other modes, employing online, distance or electronic learning [1]. Whilst not ideal, teleteaching or the delivery of live teaching via online platforms may prove to be an apt solution to the cancellations that are currently taking place. Rather than leaving students to their own devices, online teaching guides student learning and places content within the overall context of their curriculum. Currently, universities utilise lecture capture technology. However, this is limited in its interactivity and ability for students to ask questions. Additionally, outside of the current crisis many are worried that it may lead to empty lecture halls and reduced participation, and often, the missed lectures are not caught up. Perhaps it is now time for universities to consider utilising other modes of facilitating learning such as live teleteaching video conference platforms whereby student engagement and interactivity can be preserved, whilst observing appropriate COVID-19 social distancing measures.

### Teleteaching and Telemedicine

Whilst online platforms may be sufficient for students in their pre-clinical years, senior medical students who are placed in clinical environments require patient contact. Indeed, communication with and examination of patients is necessary for learning and building a diagnostic clinical thought process, for as William Osler proclaimed, ‘He who studies medicine without books sails an uncharted sea, but he who studies medicine without patients does not go to sea at all’. As an alternative to clinical placements, students at Imperial College London are being given access to an online repository of patient interview recordings and cases. Many universities have released their clinical academics to work in the National Health Service (NHS), and the acute timescale for this event has meant that drastic reorganisation has needed to be done with little time for actual teaching. However, Imperial clinicians are still delivering teleteaching through computers on hospital sites, which have seen excellent student attendance and interaction. Nonetheless, student-patient engagement is still necessary, and teleteaching does not substitute actual patient contact. Patients have a multitude of pathologies and present with varying signs and symptoms. They come with differing educational backgrounds, each presenting with a unique challenge. By not being able to engage with patients, developing key clinical skills will be more difficult. Whilst this disruption may not affect senior students’ skills, younger years are more likely to be adversely affected, as it is at this stage that their clinical foundation is set.

Rather than restricting student access to patients, telemedicine technologies may be utilised. One such approach uses tablet computers which can be cleaned between patients following appropriate infection control protocols. They can be used at sites with a high risk of COVID-19 transmission; patients can be given a tablet and isolated in an exam room. In turn, both students and physicians can communicate with these patients without risking exposure to the pathogen and wasting personal protective equipment [2]. Not only would this help clinical students to maintain and refine their diagnostic thought process but could also allow them to aid healthcare systems by reducing the burden of COVID-19 through the triage of patients. Healthcare provision through telemedicine will become the mainstream in the coming years. Indeed, studies have found that interaction with telemedicine technologies during undergraduate medical training contributes to improved core competencies, medical knowledge, overall learning and higher quality patient care [3].

Furthermore, examinations have suffered from cancellations. Students are examined regularly throughout the course, and performances in different exams often have a good correlation. In turn, examination disruptions in a single instance would not alter the predictive ability of previous exams in assessing the competency of students. As a compromise, some medical schools are turning to utilise tele-technologies in order to conduct remote assessments in an effort to ensure that final year medical students have met the required competencies before they begin to practise [4].

### Looking to the future

A key educational dilemma involves looking at the length of the epidemic. If indeed, as seems to be the case, it was set to last several months; this would lead to a substantial loss of learning time for students and probable depreciation in confidence, although the slight loss of clinical skills would likely be quickly rekindled once students are back in a clinical environment. Importantly, throughout this crisis, what will be ever-present is the use of textbooks. With the emergence of teleteaching platforms, both can be combined to fill in the gaps that would otherwise normally be learned from lecturers or clinicians on the wards. As a side note, learning should always be placed in the modern context, and great novels of the plague, such as Daniel Defoe’s Journal of the Plague Year, Manzoni’s The Betrothed, or Camus’s La Peste, can provide students with highly relevant perspectives to the current predicament we find ourselves in. This will not only illustrate why microbiologists have worried about ‘the big one’ for so long but may even motivate students to pursue a career in infectious disease and help in the prevention of futre outbreaks.

## Conclusion

As healthcare systems are set to be further stretched with the increasing burden of COVID-19, disruptions in medical education are inevitable across the world. Arrangements need to be made whereby students can retain clinical skills and knowledge. Though not without its problems, teleteaching technologies have the potential to substitute in-person lecture and clinical-based teaching, particularly during this pandemic. Such approaches may not only be necessary for effectively tackling the medical education dilemma during this current crisis but will also serve to lay the foundation for teaching during future disasters and beyond.

## Data Availability

Not applicable

## Abbreviations

NHS: National Health Service
